# Seasonal variation of *Hedruris dratini* (Nematoda) parasitizing *Hydromedusa tectifera* (Chelidae), with focus on host’s torpor state

**DOI:** 10.1186/s40850-021-00078-6

**Published:** 2021-05-01

**Authors:** Ezequiel Palumbo, María Julia Cassano, Leandro Alcalde, Julia Inés Diaz

**Affiliations:** 1 Centro de Estudios Parasitológicos y de Vectores (CEPAVE), FCNyM, UNLP, CONICET, Boulevard 120 s/n e/61 y 62, 1900 La Plata, Buenos Aires Argentina; 2 Instituto de Limnología Dr. Raúl A. Ringuelet (ILPLA), FCNyM, UNLP, CONICET, Boulevard 120 s/n e/61 y 62, 1900 La Plata, Argentina

**Keywords:** Amphipods, Feeding ecology, Hyalella, Life cycle, Nematods, South America, Turtles, Urban stream

## Abstract

**Background:**

The aim of this study was to analyze the seasonal distribution of the nematode *Hedruris dratini* parasitizing the South American Snake-necked turtle *Hydromedusa tectifera* and the amphipod *Hyalella* spp. in an urban stream. We focused on understand which strategies parasite population displays to get through the host’s hibernation period.

**Results:**

The highest prevalence and abundance of *H. dratini* were found in summer. The parasitic load was lower in winter, however there were no significant differences when it was compared with autumn and spring. Generalized linear model identified the temperature as a determining factor for the presence of parasites in turtles.

**Conclusions:**

Our results indicate that, beside turtles enter in a diapause state, the life cycle of *H. dratini* never stop throughout the year, being a continuous transmission between both the intermediate and final host throughout the year. Turtles feed and become infected with parasite larvae even in winter although with a lower ingestion rate.

## Introduction

Which factors influence the success of a parasite population? Many studies have analyzed the population dynamic of parasites in diverse kinds of host and environmental systems (e.g. [3, 4, 13, 38]). However, only few of them have dealt with this relationship from hosts that enter in a deep pause during cold months (hibernation). This last point was mostly analyzed for mammals (e.g. [10, 19, 29, 31, 32, 35]), and reptiles from North Hemisphere in a lower degree [16, 18, 40]. But how does this behavior influence the population dynamic of a parasite species if the host is a subtropical turtle that goes through a dormancy phase?

When environmental temperature decreases freshwater turtles tend to find refuge, stop feeding and diminish their metabolic rate to a minimum, entering into dormancy (torpor). This state affects nutrition, immunity, habits and dispersion, and it may last days, weeks or several months depending on the severity of climatic features [39].

The internal environment of the host where parasites inhabit is analogous to the ecological niche of a non-parasitic free-living species. Parasites can influence hosts by altering their physiology, behavior and diet [6]. Also they suffer the influence of seasonal changes, e.g., environmental variables may affect the survival of larval stages and transmission of eggs [2]. Thus, parasites have two ways to survive when environmental variables become adverse: either spending unfavorable periods outside hosts as larval stages or staying within hosts as adults but displaying adaptations to compensate the stress [12, 14].

There are few parasite records for the South American Snake-necked turtle *Hydromedusa tectifera* Cope, 1870 in Argentina, two trematodes: *Cheloniodiplostomum testudinis* (Dubois, 1936) and *Amphiorchis* sp. [24, 26], and three nematodes: *Hedruris dratini* [25], *H. orestiae* (Moniez, 1889) and *Spiroxys contortus* (Rudolphi, 1819) [23, 25]. *Hedruris dratini* (Hedruridae) is a highly prevalent nematode parasitizing the stomach of different Argentine populations of *Hyd. tectifera*. This parasite has an indirect life cycle which includes amphipods of the genus *Hyalella* Smith, 1874 as intermediate hosts [25].

In this context, the main purpose of the present work is to describe the population dynamic of *H. dratini* parasitizing *Hyd. tectifera* in an urban stream over the course of 1 year, with emphasis on understanding if parasite population reduce its activity during the host’s torpor state. Then, the following questions arise: Does the population of *H. dratini* in the Snake-neck turtle fluctuate throughout the year? If there is any variation, what factors influence these changes? Females releasing eggs when turtles are in torpor? Does turtle’s diet vary throughout the year? Are there changes in the prevalence of parasitized intermediate hosts between seasons?

We hypothesize that the population of *H. dratini* will be higher in warmer months, and will decrease in colder ones. These seasonal changes are be due to differential activity of both definitive and intermediate hosts, since lower temperatures have a direct influence on these cold-blooded animals.

The following specific objectives were proposed: (1) To analyze and compare prevalence and abundance of *H. dratini* in *Hyd. tectifera* between seasons, discriminating also between size and sex of turtles; (2) To relate the influence of weather conditions (i.e. temperature and accumulated rainfall) on prevalence of *H. dratini* in *Hyd. tectifera*; (3) To analyze seasonal variations of sex ratio and maturity rates of *H. dratini* in *Hyd. tectifera*; (4) To describe feeding habits of *Hyd. tectifera* throughout the year with emphasis on the ingestion of the amphipods *Hyalella* spp.; and (5) To analyze seasonal variations in prevalence of *H. dratini* parasitizing *Hyalella* spp.

## Materials and methods

### Sampling and study site

**Fig. 1:**
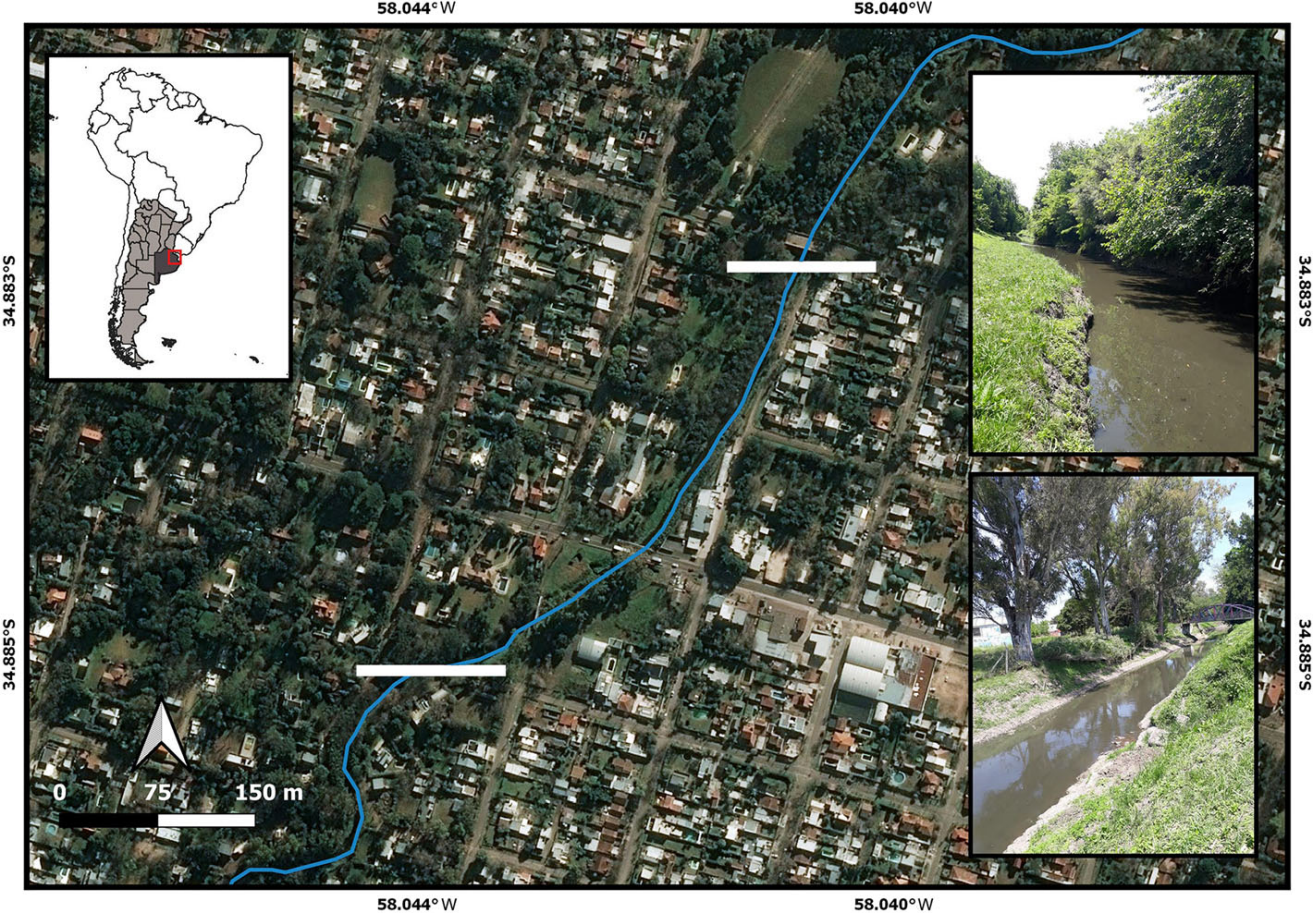
Map of the study area indicating the section of the Rodriguez stream where hosts were sampled

Samples were taken at the beginning and end of each season (summer, autumn, winter and spring) during 2018 (eight samplings in total) in the Rodriguez stream (34°53′02″ S; 58°02′30″ W, datum: WGS84): City Bell, La Plata, Buenos Aires province, Argentina (Fig. 1). The stream originates in semirural areas at the NW of the city and its middle course runs through urban areas before turning again into a rural stream that ends in the Río de La Plata Estuary by a channelized upper portion. The middle course receives sewage and garbage from surrounding houses. Therefore, this is a very disturbed stream for which physicochemical parameters and biotic characteristics were described by different authors [15, 22, 36]. Forty individuals of *Hydromedusa tectifera* were caught manually on the middle section of the stream in each season (20 at the beginning and 20 at the end of each season), totalizing 160 individuals for the four seasons. The captured turtles were taken to the laboratory in order to be: sexed (according dimorphic characters and, in case of juveniles, by penis eversion following the technique proposed by [33], weighed (W: accuracy 1 g), digital caliper measured (straight-line carapace length, SCL: accuracy 1 mm), marked (following [9]), stomach–flushed (according [21]), and fecal sampled by passing a night into individual plastic boxes. After filtering the water from the boxes, which contained feces, turtles were carried to the stream and released at the capture site. Stomach and fecal samples were kept in individual containers with 70% ethanol.

Additionally, samples of environment preys in the stream were taken in each sampling, by passing 15 times a hand net (30 cm mouth, 10 cm depth, 1 mm net). These samples were preserved in 70% ethanol and carried to the laboratory for quantification of intermediate hosts (i.e. *Hyalella* spp.) containing larval stages of *Hedruris dratini* and to know the alimentary offer (environmental availability and abundance of aquatic prey items).

Temperature and rainfall data corresponding to the sampling period were obtained from the La Plata Automatic Weather Station (Seismology and Meteorological information Department - Faculty of Astronomic and Geophysical Sciences of the National University of La Plata).

### Parasites study

Stomach and fecal samples were examined under a stereomicroscope (Leica M60®, Singapore). Found nematodes were temporarily mounted and cleared in Amman’s Lactophenol, and studied under a compound microscope (Olympus BX51®, Tokyo, Japan). All specimens were measured and counted (in summer only 50% were measured in reason of the high parasite abundance). Measurements are given in micrometers unless otherwise indicated.

A subset of 100 amphipods of each sampling were separated and clarified with Amman’s Lactophenol before observation under a stereomicroscope to search larval stages of *H. dratini*.

### Diet study

Prey items recovered from both stomach and fecal samples and also those available from the environment were classified to the lowest possible taxonomic level and counted. For both stomach and fecal samples the maximum length and maximum width of each item was measured under a stereomicroscope with measurement accessories (± 0.1 mm). Both measurements were used to estimate the volume of every item by employing the ellipsoid method of Dunham [17].

Contribution of each item to the diet was evaluated using the Relative Importance Index (RII) of [28], which formula is: RII = %OF [%V + %N], where %OF indicates the occurrence frequency (percent of the stomachs containing a particular prey item), %V is the proportion of the volume of each item in relation to the total volume of all prey items, and %N represents the numeric frequency (proportion between the number of individuals of each item and the total of individuals of all prey items). The prey item with the highest value of RII was used to rank the percentage values of the remaining items. The following RII item categories were considered: Fundamental (RII values between 75.1 and 100%), Secondary (50.1–75%), Accessory (25.1–50%), and Accidental (lower than 25%). To make RII calculation easy, overrepresentation of low abundant item categories were avoided by clustering similar item categories into more inclusive groups (e.g., several genera of aquatic coleopterans were included at family level). To calculate RII we also discriminated between larval and adult stages (e.g., for Coleoptera, Diptera, Anura) since predation upon these forms has different biological meaningful. In complement, the Shannon diversity index (H′) using the natural logarithms [37] was applied to analyze diversity of trophic items found in diet of turtle (H_d_) and availability in the stream (H_o_). The significance between H′ values was assessed using *T - test*.

### Parasite population parameters

The number (n), prevalence (P) and mean intensity (MI) of *H. dratini* were calculated as parasite population parameters following Bush et al. [8]. The term “component population”, also following Bush et al. [8], was applied. We calculated prevalence in two component populations of *H. dratini*: adults in turtles (found both in their stomach flushing and/or fecal samples), and larvae in amphipods. Also, we calculate prevalence of males and females parasitizing turtles.

### Statistical analysis

To test the different relationships, the following statistics were applied:

- Pearson’s chi-squared test, (χ^2^) was used to determine significant differences on: parasitic prevalence between recaptured and non-recaptured turtles; seasonal variations on prevalence of *H. dratini* in *Hyd. tectifera*; parasitic prevalence of *H. dratini* among size classes of *Hyd. tectifera* based on three ranges of straight carapace length of turtles (SC I: < 130 mm; SC II: 130–200 mm, and SC III: > 200 mm: see [5]); seasonal variations on prevalence of larvae of *H. dratini* in *Hyalella* sp.; seasonal variations on the sex ratio of *H. dratini*; seasonal variations on the gravid/not gravid ratio for females of *H. dratini*.

In the case that contingency tables find significant differences we proceed to make *χ*^*2*^ paired comparisons.

- One-way analysis of variance (ANOVA) was applied to find significant differences on both prevalence and size of *H. dratini* parasitizing *Hyd. tectifera* by season. Normality (Nm) and homoscedasticity (Hm) data were analyzed using an F Test with Post Hoc Tukey HSD. In cases of non-compliance with Nm or Hm, several transformations were applied depending on the case. ANOVA with Welch modification was used as omnibus test (data were transformed to obtain normality). When differences were found, Welch type T test with FDR (False Discovery Rate) control by the Benjamini - Hochberg method was used to explore variables pair to pair.
- Generalized linear model (GLM) was used to explore the relationship between prevalence of *H. dratini* with temperature and rainfall, and length of females with their state of gravidity. In the last case we employed a Binomial GLM for binary response (y = 1: female gravid, y = 0: female not gravid) that outputs the probability of a female being gravid with respect to its length. The formula is:
  $$ logit(p)=\ln \left(\frac{p}{1-p}\right)={\beta}_0+{\beta}_1L+{\beta}_2{L}^2+\varepsilon $$

where *p* is the probability that the female is gravid, *L* is the length of the female (mm) and *β* is the wanted parameter, with ε a random error term.

The same formula was applied to explore the binary response (y = 1: presence of parasite, y = 0: absence of parasite) that relates the probability (Logit) of being parasitized with respect to temperature (T) and accumulated rain (R):

$$ logit(p)=\ln \left(\frac{p}{1-p}\right)={\beta}_0+{\beta}_1R+{\beta}_2T $$

- Spearman’s correlation was applied to relate turtle size with parasites abundance.

T-test was used to compare variations in abundance of *H. dratini* between males and females of *Hyd. tectifera.*

The *p-values* lower than 0.05 were considered significant in all cases; post hoc analyses were performed with p-values adjusted by the Benjamini - Hochberg method. All analyses were carried using the R version 3.6.1, with Car and Tidyverse packages [30].

## Results

### Turtle-parasite relationships

From the 160 captured *Hydromedusa tectifera* turtles, 103 were males and 57 females. Ten turtles belonged to SC I (< 130 mm), 96 to SC II (130–200 mm), and 54 to SC III (> 200 mm). Seasonal variation on turtle sex ratios (male: female) favored males in almost all seasons: 1.1:1 (summer), 1.2:1 (autumn), 1:1 (winter), and 1.5:1 (spring). A total of 38 turtles were recaptured at one occasion from which three of them were recaptured once again.

A total of 2059 *H. dratini* (*P* = 65%; MI = 12.86) from *Hyd. tectifera* was identified, from which 1125 were males (*P* = 55%; MI = 12.36), and 934 were females (*P* = 45%; MI = 10.61) (Table 1).

**Table 1:** Detail of *H. dratini* on definitive and intermediate host

	*Hyd. tectifera*		*Hyalella* spp. (hemocoel)
	(Stomach flushing)	(Fecal samples)	
Males	521	604	62
Females	522	412	50
Infected hosts	72	66	90
Total host	160	160	800
Age category	Adult	Adult	Larvae

There were no significant differences in the prevalence of *H. dratini* between recaptured and single-caught turtles (χ2 = 0.06; df = 3; *p*-value = 0.81). For this reason, we included recaptures in all subsequent analyses.

There were not significant differences in the prevalence between turtle sex across seasons (*χ*^*2*^ = 0.36; df = 3; *p-value* = 0.95). Also, no significant differences were found in the parasite prevalence among turtle size classes (*χ*^*2*^ = 0.19; df = 2; *p-value* = 0.90), or turtle sex (*χ*^*2*^ = 0.02; df = 1; *p-value* = 0.88). The parasitic abundance did not vary (T = − 0.06; df = 97.4; *p-value* = 0.94) related to turtle sex. Finally, no relationship was found between both turtle size and number of parasites (ρ = 0.1463).

**Fig. 2:**
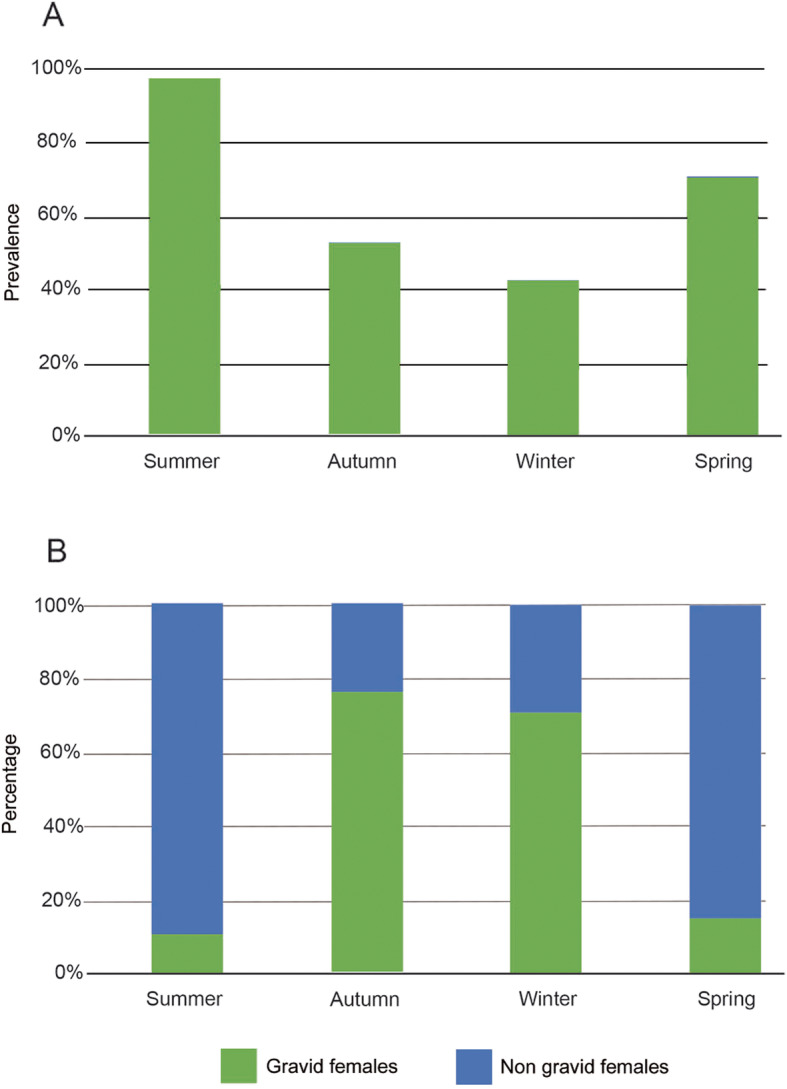
**a** Prevalence of *H. dratini* in turtles by season. **b** Relative percentage of gravid vs not gravid females of *H. dratini* in each season

The prevalence of *H. dratini* in turtles showed significant seasonal differences (*χ*^*2*^ = 30.89; df = 3; *p-value* = 8.96e-07) (Fig. 2. A), being summer different from the rest. The abundance of *H. dratini* in turtles also showed seasonal variations being summer the season which differs from the rest (*F-value* = 15.92; df = 3; *p-value* = 1.72e-07). Thus, the summer was the season with both the highest prevalence and abundance.

Once the summer identified as the key season we attempted to identify which climatic variable had the greatest impact on prevalence of *H. dratini* in turtles. The GLMs analysis between temperature and accumulate rainfall (LogLik = − 79.98; df = 3) showed that temperature is the variable that had most influence on the parasite prevalence (Table 2).

**Table 2:** Relationship between temperature and rainfall with prevalence of *H. dratini*

	*Estimate*	*Std. Error*	*p – value*
Intercept	−3.051	0.622	9.44 e-07
Rain	0.012	0.009	0.190
Temperature	0.214	0.041	1.55 e-07

The length of *H. dratini* males varied significantly among seasons [F = 9.55; df = (3; 336); *p-value* = 4.55e-06]. HSD Tukey’s test showed that summer is the season which differs from the rest by having the smallest individuals. We found similar seasonal differences in the length of parasite females [F = 21.93; df = (3; 354); *p-value* = 4.74e-13] but distinguishing the smallest sizes on summer and spring from the largest ones on autumn and winter.

Significant differences were found in the ratio of gravid: no gravid females among seasons (*χ*^*2*^ = 195.67; df = 3; *p-value* = 3.63e-42) (Fig. 2b), with a higher proportion of gravid females in cold (≈ 0.73) than in warm (≈ 0.12) seasons. All females that reached 7 mm in length were gravid. The best model to predict whether a female is gravid was the logistic one for both the presence of eggs and female length (LogLik = − 87.02; df = 2) (see Table 3).

**Table 3:** Probability of a female being gravid according to her size

	*Estimate*	*Std. Error*	*p – value*
Intercept	−26.140	4.350	1.87 e-09
Length	4.883	0.941	2.13 e-07
Length^2^	−0.201	0.050	7.71 e-05

### Amphipod-parasite relationships

**Fig. 3:**
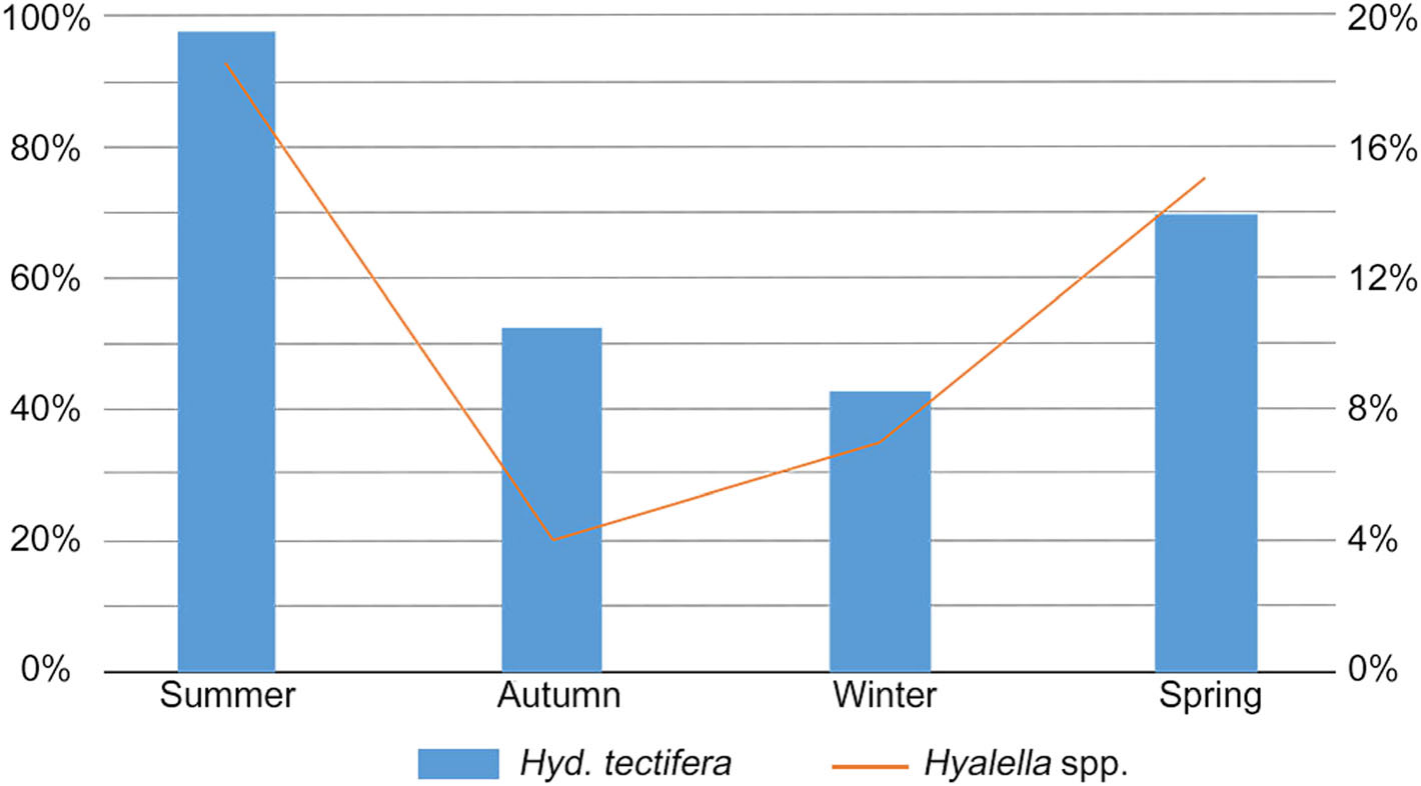
Percentage of turtles and amphipods parasitized with *H. dratini* by season

From the 800 amphipods examined, 90 were parasitized by *H. dratini* larvae located in the hemocoel (*P* = 11%, MI = 1.24) (Table 1). No other parasite species were found. Most amphipods were parasitized by only one larva but up to three parasites could be found together in the same amphipod in some cases. The prevalence of *H. dratini* larvae in amphipods was 18% in summer, 4% in autumn, 7% in winter, and 15% in spring (Fig. 3), showing significant differences between certain pairs: summer-autumn (*χ*^*2*^ = 19.63; df = 1; *p-value* = 9.39e-06), summer-winter (*χ*^*2*^ = 10.88; df = 1; *p-value* = 9.74e-04), and spring-autumn (*χ*^*2*^ = 13.65; df = 1; *p-value* = 2.09e-04).

### Turtle diet

We identified 31 prey items in the diet of *Hyd. tectifera* (Table 4). Despite of the great variety of organisms consumed by these turtles, most relevant items according to RII were: chironomid larvae (100%), amphipods (47%), terrestrial oligochaetes (21.8%), and aquatic hemipterans (12%). RII values of prey items in diet varied seasonally. Highest values in summer were: amphipods (100%) as Fundamental item, aquatic hemipterans (68%) as Secondary item, and cladocerans (35.98%) as Accessory item. In autumn, the only Fundamental item (100%) was chironomid larvae being Accidental ones the rest of the items. In winter, chironomid pupae were Fundamental prey (100%) only, followed by amphipods (39.62%), terrestrial oligochaetes (33.58%), and chironomid larvae (25.21%) as Accessory preys. Finally, the RII detected a single Fundamental (chironomid larvae: 100%), and two Secondary (terrestrial oligochaetes: 61.1%, and amphipods: 52.57%) items in spring. The total prey volume consumed by all turtles per season was: 53.92 ml (summer), 33.37 ml (autumn), 12.66 ml (winter), and 65.48 ml (spring).

**Table 4:** *Hydromedusa tectifera*’s diet detailed by season. On separate file

	Summer			Autumn			Winter			Spring		
Food item	%NF	%OF	%RII	%NF	%OF	%RII	%NF	%OF	%RII	%NF	%OF	%RII
**Annulata**												
Hyrudinea	0.065	24.39	0.11	0.106	13.51	0.04	1.309	15.63	1.06	0.128	5.41	0.03
Oligochaeta	0.253	29.27	6.80	0.118	5.41	0.73	1.800	12.50	33,5 (A)	1.574	32.43	61 (S)
**Anura**												
Larvae, unidentified family										0.043	2.70	0.01
**Artropoda, Chelicerata**												
Araneidae	0.005	2.44	0.00									
Acari, Hydrachnidia	0.005	2.44	0.00									
Acari (terrestrial), unidentified family				0.012	2.70	0.00	0.164	3.13	0.02			
**Arthropoda, Crustacea**												
Amphipoda, Hyalellidae	11.050	73.17	100 (F)	2.853	56.76	3.61	22.09	37.50	39,6 (A)	26.13	72.97	52,5 (S)
Copepoda							0.491	9.38	0.17			
Cladocera	81.065	14.63	7,3 (A)							0.851	2.70	0.06
Ostracoda	0.382	19.51	0.50	0.861	21.62	0.16						
Isopoda	0.014	7.32	0.01							0.426	8.11	0.48
**Arthropoda, Insecta, Hemiptera**												
Heteroptera, Belostomatidae	0.751	65.85	68 (F)	0.153	27.03	1.49	1.473	6.25	2.99			
Heteroptera, Corixidae	0.037	14.63	0.09	0.012	2.70	0.00						
Heteroptera, Notonectidae							0.164	3.13	0.02			
**Arthropoda, Insecta, Coleoptera**												
Adult (Dysticidae, Hydrophilidae)	0.143	24.39	1.36				0.164	3.13	0.02	0.085	2.70	0.02
**Arthropoda, Insecta, Hymenoptera**												
Formicidae	0.023	12.20	0.01	0.083	8.11	0.01				0.128	8.11	0.03
Adult, unidentified family	0.005	2.44	0.00									
**Arthropoda, Insecta, Diptera**												
Ceratopogonidae (pupa)				0.483	10.81	0.05	0.164	3.13	0.02	0.085	2.70	0.07
Chironomidae (larvae)	2.442	56.10	13.10	81.67	81.08	100 (F)	16.04	37.50	25,2 (A)	53.11	75.68	100 (F)
Chironomidae (pupa)	3.507	41.46	13.60	13.56	48.65	9.00	56.14	43.75	100 (F)	16.34	16.22	6.47
Culicidae (larvae)	0.009	4.88	0.00							0.180	5.41	0.07
Culicidae (pupa)	0.014	2.44	0.00									
Psychodidae (larvae)	0.055	9.76	0.04	0.012	2.70	0.00				0.128	2.70	0.01
Syrphidae (larvae)	0.014	4.88	0.04									
Stratiomidae (larvae)	0.009	4.88	0.00									
Adult, unidentified family	0.023	2.44	0.00	0.012	2.70	0.00						
**Arthropoda, Insecta, Odonata**												
Zigoptera larvae, unidentified family	0.046	12.20	0.02	0.047	2.70	0.00				0.128	5.41	0.02
**Arthropoda, Insecta, Thysanoptera**												
Adult, unidentified family	0.005	2.44	0.00									
**Cyprinodontiform fish**												
Anablepidae	0.023	2.44	0.07									
**Mollusca, Gastropoda**												
Ampullaridae										0.043	2.70	0.01
Hydrobiidae	0.023	9.76	0.01	0.012	2.70	0.00				0.213	10.81	0.07
Planorbidae	0.018	4.88	0.11	0.012	2.70	0.00				0.085	2.70	0.01

The H′ indexes for both the diet (H_d_) and offer (H_o_) differed significantly within each season. Summer: 0.74 H_d_ vs. 1.71 H_o_ (T = 13.03; df = 2656; *p-value* = 1.07e-37); Autumn: 0.64 H_d_ vs. 1.11 H_o_ (T = 15.41; df = 2275; *p-value* = 4.48e-53); Winter: 1.22 H_d_ vs. 0.946 H_o_ (T = 4.80; df = 1336; *p-value* = 1.75e-06); and spring: 1.22 H_d_ vs. 0.62 H_o_ (T = 16.78; df = 2162; *p-value* = 1.64e-59). In almost cases both H′ indexes differed significantly between seasons (see Table 5).

**Table 5:** Diversity of diet of analyzed turtles (H_d_, left side) and availability in the stream (H_o_, right side)

	H_o_				
		Summer	Autumn	Winter	Spring
**H**_**d**_	**Summer**		(T = 1.42 df = 3994; *p* = 0.15)	(T = 4.38 df = 1769; *p* = 1.24e-5)	(T = 12.70 df = 3123; *p* = 4.22e-36)
	**Autumn**	(T = 6.49; df = 17,469; *p* = 8.69e-11)		(T = 3.37 df = 1474; *p* = 7.57e-4)	(T = 12.18 df = 2526; *p* = 3.02e-33)
	**Winter**	(T = 11.57; df = 663; *p* = 2.3e-28)	(T = 13.54; df = 715; *p* = 2.31e-37)		(T = 6.49; df = 1409; *p* = 1.14e-10)
	**Spring**	(T = 21.38; df = 3154; *p* = 6.83e-95)	(T = 23.98; df = 3960; *p* = 8.06e-119)	(T = 0.12; df = 950; *p* = 0.89)	

## Discussion

### Parasite population dynamics

Temperature is a determinant factor for population dynamic of parasitic helminths. Van Cleave [40], observed that most parasites showed seasonal variations with a higher prevalence in warmer months in his study about acanthocephalans parasitizing freshwater turtles in North America. Our study agrees with the author’s results since both the prevalence and abundance of *H. dratini* were significantly higher in summer. But remarkably, the prevalence of *H. dratini* in winter can be considered high (i.e., 43%), despite expecting a lower result.

On other hand, Dubinina [16] observed that during hibernation of the tortoise *Testudo horsfieldii* Gray, 1844, young nematodes continued maturing at a slower rate whereas older ones die early in the inactive period in Tadzhikistan. He further stated that there is a drastic reduction in the nematode load during tortoise hibernation. In contrast, our study revealed that the presence of gravid females is higher in both autumn and winter than in warm seasons. Differences between Dubinina [16] and our study are surely due to weather conditions. Winter is substantially colder in those latitudes than in our study area in Argentina, which generates a greater impact on the land turtles increasing their dormancy periods.

Both host size and age are proposed as factors that influence the presence of parasites [18, 27]. Esch and Gibbons [18] noted that older turtles had lower parasitic loads than younger ones in their study on *Chrysemys picta* (Schneider 1783). They provided two possible explanations: (1) diet of younger turtles tends to be more carnivorous than that in older ones which are mainly herbivorous, thus increasing the chance to acquire helminth parasites; and (2) immune response is weaker in immature than in adults turtles which facilitates parasite acquisition. In contrast, *Hydromedusa tectifera* is a generalist carnivorous turtle along its entire life cycle [1, 5], and we found no significant differences in parasitic loads between size classes of the studied turtles. Therefore, these parasites do not seem to have problems to settling down either with the space (size) or the age of turtles. The settlement of parasites seems to depend on variables such as the number of intermediate hosts ingested by turtles, and perhaps the health status of turtles too, but further studies are needed to corroborate this hypothesis. We agree with Esch and Gibbons [18] in the need to monitor the diet variation of hosts among seasons to reach a better understanding of the helminth population dynamics. For this reason we have analyzed different variables in order to deal with this phenomenon, approaching the problem from an integrated perspective.

Temperature was a key factor in explaining the prevalence of *H. dratini* in this ecosystem. In this sense, the torpor state of *Hyd. tectifera* is usually interrupted during certain warmer days. In these occasions turtles leave their lethargy state and feed. Our dietary results revealed that (a) the feeding rate suffers a drop during colder months but never ceases, and (b) amphipods are also included in the winter menu of turtles although in smaller number than summer. The prevalence of amphipods infected by larvae of *H. dratini* is lower (7%) in winter but it is sufficient to ensure continuity of the parasite life cycle throughout the year. In addition, the sex ratio of *H. dratini* remains constant throughout the year being not altered by climatic seasonal variations. Females of *H. dratini* are bigger than males, a feature which was also noted in several species of the genus (e.g., [7, 20, 34]). There are differences in the average size of males and females between seasons, being larger in cold seasons and smaller in the warm ones. Also, there are proportionally more gravid females in cold months than in warm ones. This seasonal variation can be explained by the number of smaller subadults ingested during spring and summer, decreasing the average size from the component population. Those subadults grown and mature during autumn and winter, increasing the proportion of gravid females during cold seasons.

Regarding reproduction, all parasites own the potential to reproduce once inside the turtle. Different sized males were found copulating with females, and once fixed they remained coiled around them. The fact that *H. dratini* females larger than 7 mm were all gravid lead us to hypothesize two explanations in case of finding males but females larger than 7 mm were not gravid in a given host: (1) males belong to another species, like *H. orestiae* that coexist with *H. dratini* [25]; or alternatively (2) the parasite arrived at a non-appropriate host, and therefore one could predict whether *H. dratini* is suitable for that host.

### Turtle diet fluctuations

There are two works studying the diet of *Hyd. tectifera*: one by Bonino et al. [5] in which the authors studied a turtle populations in two mountain streams (Toro Muerto and Tanti) in Cordoba province; and the other by Alcalde et al. [1], who studied the population of *Hyd. tectifera* from the Buñirigo stream in Buenos Aires province, located 65 km south of the Rodriguez stream (present study). Differences observed in the diet of turtles among these localities evidently respond to characteristics of each stream. Although turtles from Cordoba showed a slightly richer diet, it was not as diverse as one would expect when compare to the diet of those turtles living in polluted streams. The Buñirigo stream is located in a rural area under the influence of human industries activity (e.g., tanneries, manufactured food), and *Hyd. tectifera* shares habitat with the turtle *Phrynops hilarii* (Duméril & Bibron 1835) which could be influencing its diet [1]. In contrast, *Hyd. tectifera* is the only turtle species in the studied portion from the Rodriguez stream but its diet may be influenced by the pollution impact caused by sewage and garbage discharges from the surrounding city.

Bonino et al. [5] observed that the most important items in the diet of *H. tectifera* in Cordoba streams were Trichoptera larvae (RII = 33.5%), fishes (RII = 30%), and Odonata naiads (RII = 25.2%), whereas in the Buñirigo stream [1] they were immature chironomids (RII = 100%), and aquatic hemipterans Corixidae (RII = 50.9%) and Belostomatidae (RII = 9.18%). In the present work the most important items were immature chironomids (RII = 100%), amphipods (RII = 47%), terrestrial oligochaetes (RII = 21.8%), and aquatic hemipterans of the Belostomatidae family (RII = 12%). Therefore, the diet is more similar to that displayed by turtles from the Buñirigo stream than that from the Cordoba population.

Previous works noted subtle changes in the specie’s diet between hot and cold months [1, 5]. These observations agree with present results since amphipods were consumed in a higher proportion than chironomids in warmest months whereas chironomids were the most chosen prey in cold seasons.

### Life cycle dynamic

According to Palumbo et al. [25], the life cycle of *H. dratini* consists of three stages: 1- free eggs in the environment are consumed by amphipods, 2- larvae develop inside amphipods until they reach the sub-adult stage, and 3- turtles become infected when feeding on amphipods and adult parasites copulate in the stomach of turtles before females release eggs to complete the cycle. Casalins et al. [11] demonstrated that amphipods infested by larvae of *Hedruris suttonae* Brugni and Viozzi [7] display high photophilic levels. Highly photophilic amphipods are more exposed to be ingested than uninfected ones as they tend to swim freely. Thus, this anomalous behavior should be a putative explanation also for the successful life cycle of *H. dratini*, nematode capable to infect a huge number of amphipods, and consequently, turtles. As the number of infected turtles increases, more parasite eggs are released into the environment, resulting in a large number of infected amphipods continuing the cycle.

## Conclusions

Callait and Gauthier [10] described four strategies that parasites of marmots adopt to survive in winter: 1- to overwinter in the intermediate host, 2- resistant eggs on the soil, 3- larval migration out of gut with diapauses, and 4- to remain inside the host as an adult in what they called co-hibernation. Turtles studied here do not hibernate but they instead have a dormancy period that influence the presence of parasites, resulting in lower prevalence and abundance. However, the population of *H. dratini* persist throughout the year in turtles, being a continuous transmission between both the intermediate and final host.

Temperature affects some aspects of the life cycle of *H. dratini* by modifying the behavior of its hosts, increased temperature makes turtles more active, which feed more frequently and therefore consume more parasitized amphipods. Also, amphipod population is bigger in spring and summer, and at the same time the prevalence of *H. dratini* in amphipods is also higher in cool seasons. Consequently, the population of *H. dratini* reaches a peak during summer and declines in winter but its prevalence remains over 40%, allowing a quick recover when temperature starts rising. It is possible because the winter in the region is not as harsh as in other areas, allowing turtles to continue feeding and parasites to continue dispersing themselves.

The study of parasite-host-environment relationships allows us to better understand the dynamics of the parasite populations providing valuable information about biotic and abiotic patterns that affect their actual and futures distributions.

## Data Availability

The datasets generated and/or analyzed during the current study are not publicly available due the data are part the EP Doctoral Thesis that has not yet been published but are available from the corresponding author on reasonable request.
